# Healthcare costs and utilization for Medicare beneficiaries with Alzheimer's

**DOI:** 10.1186/1472-6963-8-108

**Published:** 2008-05-22

**Authors:** Yang Zhao, Tzu-Chun Kuo, Sharada Weir, Marilyn S Kramer, Arlene S Ash

**Affiliations:** 1 Eli Lilly and Company, Indianapolis, IN, USA; 2 DxCG, Inc, Boston, MA, USA; 3 Center for Health Policy and Research/Department of Family Medicine and Community Health, University of Massachusetts Medical School, Shrewsbury, MA, USA; 4 Partnership for Healthcare Excellence, Boston, MA, USA; 5 Health Care Research Unit, Section of General Internal Medicine, Department of Medicine, Boston University School of Medicine, Boston, MA, USA; 6 Formerly of DxCG, Inc, Boston, MA, USA

## Abstract

**Background:**

Alzheimer's disease (AD) is a neurodegenerative disorder incurring significant social and economic costs. This study uses a US administrative claims database to evaluate the effect of AD on direct healthcare costs and utilization, and to identify the most common reasons for AD patients' emergency room (ER) visits and inpatient admissions.

**Methods:**

Demographically matched cohorts age 65 and over with comprehensive medical and pharmacy claims from the 2003–2004 MEDSTAT MarketScan^® ^Medicare Supplemental and Coordination of Benefits (COB) Database were examined: 1) 25,109 individuals with an AD diagnosis or a filled prescription for an exclusively AD treatment; and 2) 75,327 matched controls. Illness burden for each person was measured using Diagnostic Cost Groups (DCGs), a comprehensive morbidity assessment system. Cost distributions and reasons for ER visits and inpatient admissions in 2004 were compared for both cohorts. Regression was used to quantify the marginal contribution of AD to health care costs and utilization, and the most common reasons for ER and inpatient admissions, using DCGs to control for overall illness burden.

**Results:**

Compared with controls, the AD cohort had more co-morbid medical conditions, higher overall illness burden, and higher but less variable costs ($13,936 s. $10,369; Coefficient of variation = 181 vs. 324). Significant excess utilization was attributed to AD for inpatient services, pharmacy, ER visits, and home health care (all p < 0.05). In particular, AD patients were far more likely to be hospitalized for infections, pneumonia and falls (hip fracture, syncope, collapse).

**Conclusion:**

Patients with AD have significantly more co-morbid medical conditions and higher healthcare costs and utilization than demographically-matched Medicare beneficiaries. Even after adjusting for differences in co-morbidity, AD patients incur excess ER visits and inpatient admissions.

## Background

Alzheimer's disease (AD) is a progressive, irreversible neurodegenerative disorder with high social and economic costs. Currently, an estimated 5.1 million Americans have AD, 4.9 million of them over the age of 65 [1]. Alzheimer's disease affects 13% of people over age 65 and nearly half of those over age 85, accounting for 50 to 70% of all dementia cases [1]. By 2050, 11.6 to 16 million Americans may have AD [2]. With the expected increase in AD cases, medical costs for Medicare beneficiaries with AD are expected to increase from $91 billion in 2005 to $160 billion in 2010 [3]. Understanding what contributes to health care costs and utilization among AD patients should help health plans develop effective disease management protocols.

Prior studies [4-16] on costs and utilization associated with AD in the US have several limitations: 1) most use data collected prior to 2000 which do not capture current treatment patterns [4-12,15,16]; 2) several "contaminate" their definitions of AD cohorts by including diagnosis codes of non-AD dementias in their claims data analyses [4-6,8,12]; 3) several present only aggregated total cost data or omit pharmacy costs [4,9,10,12]; and 4) most rely on the presence of, at most, a small subset of co-morbidities to account for the effect of differences in disease burden between cases and controls [4-16].

In addition, no previous research has explored which medical conditions lead to inpatient admissions and emergency room (ER) use for patients with AD or estimated how AD affects such utilization beyond what would be expected based on the presence of co-morbidities.

In this study we sought to identify differences in direct healthcare costs and utilization, and common reasons for ER visits and inpatient admissions between Medicare beneficiaries with an AD diagnosis and controls, after comprehensively adjusting for the presence of other co-morbidities. Using a large, US administrative claims database, we examined 2004 direct healthcare costs and utilization for individuals aged 65 and above in each cohort with comprehensive medical (including claims paid by Medicare) and pharmacy claims. Illness burden was measured by a comprehensive disease classification and scoring system and used to produce estimates of the marginal effect of AD on inpatient, ER, pharmacy and other utilization and costs. Reasons for ER visits and inpatient admissions were also examined for both AD and control cohorts, and logistic regression was used to assess the contribution of AD to the most common reasons for ER visits and hospitalizations controlling for differences in overall illness burden. Findings from this research may help healthcare providers and health plans in the US develop protocols to better manage patients with AD.

## Methods

### Study Sample

Data were obtained from the MEDSTAT MarketScan^® ^Medicare Supplemental and Coordination of Benefits (COB) Database for 2003 and 2004. These data contain information about Medicare beneficiaries across the US, age 65 and older, with employer-sponsored supplemental insurance including pharmacy benefits. The eligible study population was comprised of 627,775 individuals with 12 months of enrollment in 2003, at least one month of enrollment in 2004, and comprehensive inpatient, outpatient, and pharmacy claims. Coverage for skilled nursing facility care is limited under Medicare, and generally long term nursing home care is not covered by employer-sponsored supplemental insurance. Thus, the cost and utilization data presented here do not include care provided in skilled nursing facilities and nursing homes. Because data used in this study were purchased from a third party which had removed identifying information prior to its release, institutional review board (IRB) and similar approvals were neither needed nor sought.

From this population we selected 25,109 individuals with AD and three times as many demographically-matched controls. The AD cohort contained all patients with at least one non-laboratory claim with an AD diagnosis (ICD-9-CM code 331.0) or at least one filled prescription for an AD-specific medication (tacrine, donepezil, galantamine, rivastigmine, or memintine) in 2003. The control cohort excluded both those with AD using the above criteria and those with any other form of dementia (ICD-9-CM codes 290.xx, 294.1x, 294.8x, 331.1x-331.9x, 797.xx) recorded at any time in either 2003 or 2004. Because a diagnosis of AD is not always accurate, it is likely that the AD cohort includes some number of people with dementia arising from other causes, such as vascular dementia.

We used propensity scores to construct the demographically matched control cohort. First we predicted the probability of "propensity to have" AD using logistic regression. Explanatory variables included: 1) age as of January 1, 2003, 2) age squared, 3) gender, 4) geographic region, and 5) months of health plan enrollment in 2004. The data were ranked by the probability of having AD (from highest to lowest), and then partitioned into twenty quantiles (with 5% of the population in each). Within each 5% quantile, eligible individuals were randomly selected to match each individual in the AD cohort using a 3 to 1 ratio.

### Controlling for Illness Burden

We measured overall illness burden using DxCG, Inc.'s RiskSmart™ software, which includes the Diagnostic Cost Groups (DCGs) model. This validated, diagnosis-based classification system [17] organizes over 15,000 ICD-9-CM codes into 781 highly homogeneous clinical categories (DxGroups), which are further clustered into 184 Condition Categories (CCs) that encompass similar medical conditions with similar expected costs [18]. The software further organizes the CC information for each person into a 184-variable vector of "hierarchical CCs" (HCCs), where the presence of a more serious manifestation of a disease causes clinically-less-relevant conditions to be "zeroed out." For example, "chronic obstructive lung disease" dominates "cold" [18].

We predicted 2004 health care costs for the eligible study population using HCCs from the 2003 diagnoses, age, and sex. The CC for AD was excluded from the estimation so that the AD and control cohorts would have comparable predicted costs after controlling for demographics and all co-morbidities other than AD in 2003.

Predicted costs were then normalized (multiplied by the appropriate constant) to create prospective relative risk scores (RRSs) that average 1.00 in the eligible study population.

### Analyses

Demographics such as age, gender, and region were used to characterize the AD cohort and controls. We also calculated mean duration of health plan enrollment in 2004, the number of unique co-morbid conditions, and the RRS for non-AD illness burden.

Health care costs and utilization were calculated for 2004 by place of service, including physician office visits, outpatient hospital services, ER visits, inpatient services, home health care, and pharmacy. All costs included deductibles, copayments, coinsurance, and coordination-of-benefits payments. For partial year enrollees, utilization and expenditures were annualized (actual totals divided by percentage of year enrolled). Total health care costs were compared using the mean, computed t-tests of the differences in means, standard deviation (SD), cost dispersion as measured by the coefficient of variation (CV) and cost distribution by place of service. Health care utilization was evaluated by examining percentages of users, the mean cost per member per year and, where applicable, the number of encounters (visits) per member per year.

Regression was used to estimate AD's independent effect on overall costs and utilization. Specifically, Weighted Least Squares (WLS) regressions were used to assess the marginal contribution of AD to 2004 costs and health care utilization, weighted by the fraction of time enrolled in 2004. Explanatory variables included: 1) an indicator variable (0/1) identifying individuals in the AD cohort; 2) the RRS, as a measure of total non-AD illness burden; and 3) RRS squared. The coefficient associated with the AD indicator measures the extent to which AD contributes to excess healthcare costs or utilization after controlling for differences in overall illness burden.

The most common reasons for inpatient admissions and for ER visits were identified by mapping the first diagnosis from each claim into a DxGroup category. We also compared the prevalence per 10,000 persons of ER visits and inpatient admissions between the AD cohort and controls. We assessed the marginal contribution of AD to ER visits and inpatient admissions controlling for difference in overall illness burden via logistic regressions using the same predictors in WLS regressions as noted above.

All analyses were performed using SAS software (version 9.1, SAS, Cary, NC).

## Results

As constructed, the control group did not differ from the AD cohort with respect to age, sex, regional distribution, or mean length of eligibility in 2004 (Table 1). However, the AD cohort had more co-morbidities (mean of 8.1 CCs vs. 6.5, p < 0.001), and a greater burden of non-AD illness (mean RRS of 1.23 vs. 1.04, p < 0.001).

**Table 1:** Demographics, Health Plan Enrollment, and Illness Burden

	AD Cohort	Control Group
	
	N = 25,109	N = 75,327
Characteristic		
Age, Mean (SD)*	80.1 (6.5)	80.1 (6.6)
Age, n (%)		
65 – 69	1,529 (6.1)	4,613 (6.1)
70 – 74	3,482 (13.9)	10,330 (13.7)
75 – 79	6,345 (25.6)	19,641 (26.1)
80 – 84	7,326 (29.2)	21,471 (28.5)
85 – 89	4,654 (18.5)	13,428 (17.8)
90+	1,683 (6.7)	5,844 (7.8)
Female, N (%)	15,473 (61.6)	47,082 (62.5)
Regional distribution, N (%)		
Northeast	2,986 (11.9)	10,783 (14.3)
North Central	8,710 (34.7)	25,725 (34.2)
South	9,178 (36.6)	24,028 (31.9)
West	4,185 (16.7)	14,534 (19.3)
Unknown	50 (0.2)	257 (0.3)
Months of health plan enrollment in 2004, Mean (SD)	11.1 (2.51)	11.2 (2.34)
Enrolled all 12 months of 2004, N (%)	21,380 (85.2)	65,949 (87.6)
Number of Comorbidities, Mean (SD)^†§^	8.1 (5.5)	6.5 (5.0)
Illness burden score, Mean (SD)^‡§^	1.23 (0.80)	1.04 (0.77)

* Age as of 01 Jan 2003 displayed as mean ± standard deviation (SD)

† Diagnostic Cost Group (DCG) Condition Categories (CCs), excluding AD.

‡ Prospective Relative Risk Score (RRS) excluding AD, normalized to 1.0 for the eligible study population.

§ Statistically significant differences between the two groups at p < 0.001.

Annualized costs and utilization by place of service are summarized in Table 2. The vast majority of AD patients (97%) and controls (91%) used some healthcare services in 2004. Rates of ER visits (41% vs. 27%), inpatient hospital stays (30% vs. 20%), and home health care (7% vs. 4%) were about 50% higher for AD patients than for controls. Controlling for overall illness burden, the excess utilization attributed to AD for inpatient services, ER visits, and home health care were all significant (p < 0.05). However, the AD cohort used fewer physician office visits and outpatient hospital services (both p < 0.05). Differences in costs attributed to AD were statistically significant (p < 0.05) for all categories except home health care. Spending in the AD cohort was higher for all but two categories of services (outpatient services and office visits) compared to controls, but AD spending was always less variable (coefficient of variation (CV) was lower). Excess pharmacy costs associated with AD were $1,711, more than twice that of any other expense category (p < 0.05).

**Table 2:** 2004 Annualized Health Care Costs and Utilization by Type of Service

	AD			Controls			Contribution of AD^†^	
			
	% of Cohort Using	Mean Visit Days	Mean Cost $ (CV)*	% of Cohort Using	Mean Visit Days	Mean Cost $ (CV)*	Visit Days^†^	Cost $ ^†^
Inpatient	30	3.38	5,094 (419)	20	1.93	4,014 (753)	1.14^‡^	671^‡^
Pharmacy	94	--	4,056 (77)	85	--	2,169 (174)	--	1,711^‡^
Hospital outpatient	61	2.61	1,252 (298)	58	2.51	1,412 (384)	-0.30^‡^	-366^‡^
Physician office	79	8.46	1,100 (237)	81	9.97	1,508 (270)	-2.86^‡^	-648^‡^
Emergency room	41	1.04	335 (280)	27	0.64	196 (530)	0.28^‡^	107^‡^
Home health	7	0.24	32 (1,001)	4	0.15	27 (3,191)	0.05^‡^	0.37
Other	56	--	2,068 (353)	37	--	1,043 (571)	--	789^‡^
								
Total Utilization	97	--	13,936 (181)	91	--	10,369 (324)	--	2,307^‡^

* CV = Coefficient of Variation = 100*SD/Mean

† Coefficient of the AD indicator from the weighted least square regression for annualized costs, or utilization within each setting controlling for overall illness burden (RRS excluding AD and RRS squared).

‡ Statistically significant effects attributed to AD at p < 0.05.

The 10 most common reasons for ER visits among individuals belonging to the AD or control cohorts, and their rates and comparative prevalence, are shown in Table 3. The AD and control cohorts shared 6 of their 10 most common reasons, and their top two reasons (contusion/superficial injury and chest pain) were the same. The AD cohort had higher raw ER use rates than controls for all 14 reasons listed, and significantly higher risk-adjusted use rates for 11 of them with odds ratios (ORs) ranging as high as 5.85 for stupors and other states of altered consciousness. Only one of the 14 reasons shown (heart failure) had risk-adjusted ER use rate lower for AD patients than for controls (OR = 0.87, p < 0.05). The odds of an individual in the AD cohort having an ER visit for any reason was 74% greater than for controls.

**Table 3:** Top 10 Reasons for ER Visits by Cohort

	AD	Control	Odds Ratio*
	Rate per 10,000 (Rank within cohort)†		

Contusion/superficial injury	**679 (1)**	**270 (2)**	2.23^‡^
Chest pain	**480 (2)**	**353 (1)**	1.16^‡^
Syncope and collapse	**333 (3)**	**115 (10)**	2.64^‡^
Open wound except eye and lower arm	**316 (4)**	**115 (11)**	2.54^‡^
Cystitis, other urinary tract infections	**306 (5)**	103 (13)	2.69^‡^
Other general symptoms	**300 (6)**	**130 (7)**	2.07^‡^
Other and unspecified pneumonia	**288 (7)**	**145 (5)**	1.74^‡^
Abdominal/pelvis symptoms	**279 (8)**	**219 (3)**	1.10
Stupor/altered consciousness/trans global amnesia/febrile convulsions	**264 (9)**	41 (37)	5.85^‡^
Disorders of fluid/electrolyte/acid-base balance, e.g., dehydration	**252 (10)**	94 (16)	2.36^‡^
Heart failure	192 (14)	**181 (4)**	0.87^‡^
Stomach/intestinal disorders/symptoms, except obstruction, ulcer, and hemorrhage	223 (12)	**136 (6)**	1.43^‡^
Arthropathy/joint disorders, derangements, joint pain/stiffness, excluding gout	193 (13)	**122 (8)**	1.49^‡^
Nonspecific backache and other back/neck pain/disorders	141 (20)	**121 (9)**	1.05
			
Any ER visit	10,413	5,733	1.74^‡^

* Odds ratio of the AD indicator from the logistic regression predicting any ER visit controlling for overall illness burden (RRS excluding AD and RRS squared).

† 10,000 times the number of ER visits divided by number of individuals in the cohort. Bold text designates reason in the top ten.

‡ Statistically significant AD effect at p < 0.05.

Analogous to the previous table, Table 4 compares utilization for the top 10 most common reasons for inpatient admission for each cohort. Only 3 of the top reasons (hip fracture, other and unspecified pneumonia, and cystitis/other urinary tract infections) were shared. Even after controlling for illness burden, patients in the AD cohort were more likely to be hospitalized for most of the reasons listed, including pneumonia, infections, syncope and hip fracture. However, AD patients were less likely to be admitted to the hospital due to heart failure or coronary atherosclerosis and other coronary ischemic heart disease (all p < 0.05) compared with those in the control cohort. The only other reason for hospital admission that was less likely in the AD cohort was for osteoarthritis of lower leg (knee), typically related to knee surgery. The odds for an inpatient admission were 55% greater for AD patients than for controls.

**Table 4:** Top 10 Reasons for Inpatient Admission by Cohort

	AD	Control	Odds Ratio*
	Rate per 10,000 (Rank within cohort)†		

Other and unspecified pneumonia	**229 (1)**	**133 (2)**	1.50^‡^
Femoral (hip) fracture	**209 (2)**	**88 (4)**	2.32^‡^
Cystitis, other urinary tract infections	**161 (3)**	42 (13)	3.46^‡^
Heart failure	**150 (4)**	**158 (1)**	0.78^‡^
Cerebral degeneration/Alzheimer's disease	**142 (5)**	-	infinite
Disorders of fluid/electrolyte/acid-base balance, e.g., dehydration	**118 (6)**	49 (11)	2.16^‡^
Septicemia (blood poisoning)/shock	**118 (7)**	14 (39)	2.77^‡^
Syncope and collapse	**100 (8)**	33 (30)	2.85^‡^
Aspiration pneumonia	**100 (9)**	18 (16)	5.36^‡^
Pre-cerebral or cerebral arterial occlusion with infarction	**84 (10)**	48 (12)	1.74^‡^
Coronary atherosclerosis and other chronic ischemic heart disease (CAD)	70 (13)	**111 (3)**	0.53^‡^
Acute myocardial infarction, initial episode of care	83 (11)	**81 (5)**	0.92
Atrial arrhythmia	56 (16)	**62 (6)**	0.83
Osteoarthritis of lower leg (knee)	27 (29)	**62 (7)**	0.39^‡^
Emphysema/chronic bronchitis, 18+	65 (14)	**55 (8)**	1.02
Gastrointestinal hemorrhage, except peptic ulcer and anal/rectal	78 (12)	**55 (9)**	1.22^‡^
Chest pain	54 (17)	**50 (10)**	0.94
			
Any inpatient admission	3,796	2,408	1.55^‡^

* Odds ratio of the AD indicator from the logistic regression predicting any inpatient admission controlling for overall illness burden (RRS excluding AD and RRS squared).

†10,000 times the number of inpatient admissions divided by number of individuals in the cohort. Bold text designates reason in the top ten.

‡ Statistically significant AD effect at p < 0.05.

## Discussion

Our study is the first to examine the most common medical conditions among AD patients that lead to inpatient admissions and ER use, and to contrast AD patients' co-morbid diseases and utilization with those of a demographically-matched control group. Earlier studies accounted for just a few co-morbidities in seeking to isolate AD's influence on costs and utilization. Using a comprehensive co-morbidity assessment for AD patients and a demographically-matched control group, we have achieved credible estimates of the independent effect of AD on healthcare costs and utilization. Individuals in the AD cohort had more unique co-morbid medical conditions and higher overall illness burden than those in the control cohort. Use of services was greater for AD patients, with far more inpatient, ER, and home health encounters. Mean excess cost attributed to AD, even after controlling for the greater overall burden of illness, was $2,307.

The AD cohort had considerably higher pharmacy costs [7,8,13,14] and total health care costs [4,7,8,11-13] than seen in previous studies. The findings establish the need to better understand pharmacy management practices for AD patients given pharmacy's large contribution to their elevated costs. Compared with demographically-matched controls, AD patients had significantly higher but less variable costs. So although patients with AD were costly, their costs were more predictable than those in the control cohort.

In our study, AD patients on average had longer hospital days and more visits than controls for all utilization categories except physician office visits. Overall prevalence rates for ER visits and inpatient admissions were significantly higher for AD patients. Closely managing hospitalization as well as ER visits may have significant impact on health care resource use in AD.

Alzheimer's disease complicates the management of an elderly population with significant co-morbid disease. Patient non-cooperation, inability to communicate, less frequent office visits, and caregiver burden may all contribute to "simple" medical problems escalating into hospital admissions or ER visits for reasons such as pneumonia, dehydration and septicemia. Dehydration, for example, may precipitate other medical problems, including cystitis, electrolyte imbalances, contusions, and hip fractures. However, it is unclear if dehydration requiring ER care or hospitalization is really more common in AD patients; an alternative explanation is that "dehydration" is used to code admissions requested by stressed caregivers in the absence of clear clinical symptoms.

Alzheimer's patients have many co-morbid medical problems and use multiple medications [14,19,20]; they may be prone to harm themselves [13]. The use of multiple medications raises the risk of adverse drug reactions and drug-drug interactions, and complicates medication compliance [21]. Polypharmacy, especially in the elderly population, is associated with adverse drug reactions [22-24], which occur in 5–10% of hospital inpatient admissions and increase hospital stays and costs [25]. All these factors, especially when combined with impaired cognition, could contribute to the observed increase in hospitalizations for hip fracture and syncope for AD patients. These findings suggest opportunities for improvement through case management to address AD patients' co-morbidities, specifically through medication reviews.

Differences in disease prevalence also lead to higher rates of hospitalization (most prominently, hospitalizations for AD itself). However, differences in co-morbidities do not explain AD patients' lower use of hospitalizations for heart problems since heart problems were similarly common in the two cohorts. This may be due to reduced awareness of (non-obvious) heart problems or because heart problems are treated less aggressively in AD patients. For example, ER visits for "chest pain" were more common in patients with AD compared to controls, although hospital admissions were less common.

Our study has several limitations. First, we examined Medicare-eligible individuals with employer-sponsored supplemental insurance, mostly from large companies whose active or former employees do not necessarily represent the general population of Medicare beneficiaries. This may contribute to the relatively low (4%) AD prevalence in this elderly cohort. Second, AD patients in our study were identified via diagnoses in administrative claims. Thus, some non-AD patients may be in our AD cohort (due to a false AD diagnosis) while some people with AD will be excluded (due to either a lack of any AD diagnosis or to misclassification, for example, as vascular dementia). Thirdly, the costs provided in our data do not capture care provided in skilled nursing facilities or nursing homes, therefore our analysis may underestimate the total direct healthcare costs of AD. Fourthly, our claims data do not have information on the duration or severity of AD, which is significantly related to healthcare cost and utilization [7,26]. Although we controlled for differences in overall co-morbidities in our analysis, we could not control for disease severity. Fifthly, our data do not include information on living situation (e.g., home versus institution), which may also affect healthcare costs and utilization. Finally, although research has indicated that the indirect costs of AD are substantial [1], this study focused only on direct healthcare costs.

## Conclusion

Compared to a demographically-matched control cohort, AD patients had significantly more co-morbid medical conditions and higher overall illness burden. Even after adjusting for differences in overall illness burden, people with AD incurred markedly more health care costs than their age-matched peers. The greater predictability of AD spending and more frequent, more costly, and different use of hospitals, suggests opportunities for improvement. Significantly increased financial exposure to AD with the expansion of prescription drug coverage under Medicare increases the pressure on health plans to develop more effective management protocols for AD patients.

## Competing interests

YZ is employed by Eli Lilly and Company, and owns company stocks. T–CK and ASA are affiliated with DxCG, Inc. SW and MSK were previously affiliated with DxCG, Inc.

## Authors' contributions

YZ conceived the study, participated in the design, and drafted the manuscript. T–CK performed the data analysis and helped draft the manuscript. MSK acquired the data, participated in the design and coordination of the study and helped draft the manuscript. SW participated in the study design, performed the data analysis and helped draft the manuscript. ASA participated in the design and coordination of the study and helped draft the manuscript. All authors read and approved the final manuscript.

## Pre-publication history

The pre-publication history for this paper can be accessed here:


